# Investigating the effect of cholinergic and adrenergic blocking agents on maternal-fetal heart rates and their interactions in mice fetuses

**DOI:** 10.1242/bio.058999

**Published:** 2022-04-13

**Authors:** Ahsan H. Khandoker, Maisam Wahbah, Chihiro Yoshida, Yoshiyuki Kasahara, Kiyoe Funamoto, Kyuichi Niizeki, Yoshitaka Kimura

**Affiliations:** 1 Health Engineering Innovation Center (HEIC), Department of Biomedical Engineering, Khalifa University, Abu Dhabi 127788, United Arab Emirates; 2 Department of Maternal and Fetal Therapeutics, Tohoku University Graduate School of Medicine, Sendai, Miyagi, 980-8575, Japan; 3 Department of Maternal and Child Health Care Medical Science, Tohoku University Graduate School of Medicine, Sendai, Miyagi, 980-8575, Japan; 4 Department of Biosystems Engineering, Graduate School of Science and Engineering, Yamagata University, Yonezawa, Yamagata, 992-8510, Japan

**Keywords:** Autonomic regulation, Autonomic blockade, Fetal and maternal heart rate, Heart rate variability, Pregnant mice

## Abstract

This study examines the role of autonomic control of maternal and fetal heart rate variability (MHRV and FHRV) and their heartbeats phase coupling prevalence (CP_heartbeat_) in mice. The subjects are divided into three groups: control with saline, cholinergic blockade with atropine, and β-adrenergic blockade with propranolol. Electrocardiogram signals of 27 anesthetized pregnant mice and 48 fetuses were measured for 20 min (drugs were administered after 10 min). For the coupling analysis, different maternal heartbeats were considered for one fetal beat. Results show that saline infusion did not produce any significant changes in MHRV and FHRV, as well as CP_heartbeat_. Atropine increased maternal HR (MHR) and decreased MHRV significantly without any considerable effect on fetal HR (FHR) and FHRV. Propranolol infusion did not produce any significant changes in MHR and MHRV, but significantly decreased FHR and increased FHRV. Moreover, atropine had led to a decrease in CP_heartbeat_ when considering two and three maternal beats, and an increase for four beats; while propranolol resulted in a decrease for two heartbeats, but an increase for four and five beats. The proposed approach is useful for assessing the impact of maternal autonomic modulation activity on fetal distress and obstetric complications prevalent in pregnant mothers.

## Abstract (precis)

**Summary:** Autonomic development of fetal mice is analyzed through electrocardiography. Saline infusion does not alter maternal and fetal heart rate variation and coupling significantly. Atropine increases maternal heart rate, while propranolol lowers fetal heart rate.

## INTRODUCTION

Genetic manipulation of mice as a substitute for human models is a common technique employed to understand the nervous system and underlying diseases (Kaufman, 1992). Maternal factors that influence fetal development can be evaluated with the mice model. The intuitive question “How can research findings in mice models be extrapolated to human physiology?” has attracted our interest. We have reported in our previous studies (Khandoker et al., 2016, 2017, 2018, 2020) that pregnant mice models pave the way for a better understanding of the embryonic nervous system development. A single mouse embryo cell requires 3 weeks to become a fully-grown newborn (Kaufman, 1992). Since humans and mice share many genes, valuable information can be obtained from studying mice models to understand how a human embryo matures over time (Keller et al., 1996). Correlations between congenital heart defects and early embryonic mortality (Rossant, 1996) have called for new studies and technologies that would further develop the research in the area of cardiac forms and functions in the utero. During fetal mice development, most of the cranial nerve ganglia and cranial nerves were reported to be readily identifiable by embryonic day (E)15.0 of gestation due to their large size (Chen et al., 2017). The first sign of cardiac innervation during gestation were found in the dorsal mesocardium at E10.5 in mice (Hildreth et al., 2007). In a previous study (Thomas et al., 1995), fetal mice from E9.5 to E13.5 were found to be responsive to norepinephrine and catecholamines. The fact that cardiac cells are able to react to early administration of catecholamines indicates that adrenergic receptors are present and fully functional during the development of sympathetic innervation. Muscarinic cholinergic receptors were found to be widespread in nervous tissue and smooth muscle of 17- and 18-day-old mice fetuses (Lammerding-Koppel et al., 1995).

Human fetal health conditions and development are usually assessed using crown-rump length measurement, and standard ultrasonography techniques (The American College of Obstetricians and Gynecologists, 2017). While echocardiography is mainly performed to assess the heart's structure (size, thickness, and pumping chambers) (Gibbs et al., 2016; Sun et al., 2018), electrocardiography yields a non-invasive way to monitor the autonomic nervous system and its functions by peak-to-peak variation measurements (Minato et al., 2018). This vital information cannot be obtained using the relatively expensive ultrasound echocardiography.

Short- and long-term beat-to-beat heart rate variability (HRV) analysis performed on electrocardiogram (ECG) (such as the mean level, standard deviation, and the square root of the mean squared differences of successive interbeat intervals) (Task Force of the European Society Organization Electrophysiology, 1996) were reported to be associated with parasympathetic (cholinergic) and sympathetic (adrenergic) nervous system activities, respectively (Elghozi and Julien, 2007). We developed and demonstrated in our previous study (Khandoker et al., 2016) the fetal electrocardiography system to monitor and estimate beat-to-beat heart rate (HR) and its variability parameters to assess fetal mice cardiac autonomic nervous system development in the utero. Recently, a number of studies have focused on analyzing the synchronization between fetal and maternal cardiac systems and have reported episodic phase coupling between these two oscillatory processes (Marzbanrad et al., 2015; Van Leeuwen et al., 2009, 2014). Short-term fetal HRV (FHRV) estimated by the root mean square of successive differences between normal heartbeats (RMSSD) of fetal HR (FHR) was found to be positively correlated (*P*<0.05 was considered to be significant) with coupling strengths between maternal to fetal HRs (Marzbanrad et al., 2015). This could indicate that autonomic tone played a role in determining heartbeats phase coupling prevalence. Interestingly enough, no studies have yet reported the mechanism of maternal HR (MHR) and FHR regulation and interaction by the autonomic nervous system in fetal mice.

The scope of this work is to investigate the role of autonomic control of MHRV and FHRV in time and frequency domains, and their heartbeats phase coupling prevalence (CP_heartbeats_) in pregnant mice models by blocking cholinergic and β-adrenergic nerve activities with atropine and propranolol, respectively.

## RESULTS

An experimental investigation has been carried out by using pregnant mice to explore the influences of cholinergic and β-adrenergic blockades on maternal and fetal HR regulation, interaction, and their phase coupling prevalence for different heartbeat ratios. Table 1 summarizes the results of the conducted experiments in this study.

**Table 1.**
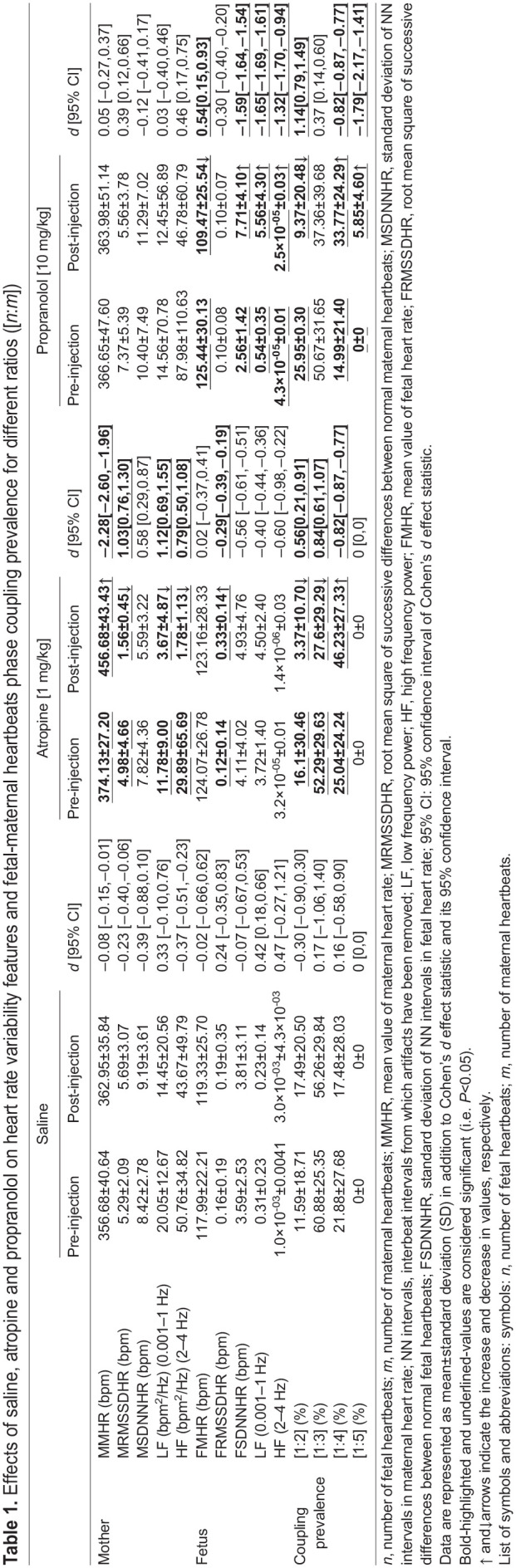
Effects of saline, atropine and propranolol on heart rate variability features and fetal-maternal heartbeats phase coupling prevalence for different ratios ([*n*:*m*])

### MHR and FHR variability

Saline infusion did not produce any significant changes in MHRV and FHRV features (Table 1). In addition, most of the associated Cohen's *d* values show small effects that are statistically insignificant. Atropine injection resulted in a significant variation in MMHR (increase), as well as to some MHRV features (including MRMSSDHR, LF and HF power components of MHR) (decrease). Interestingly, neither FMHR nor FSDNNHR is affected by atropine infusion. On the other hand, propranolol did not cause any changes in MHR and its HRV. However, FMHR significantly decreases and some FHRV features (including FSDNNHR, LF and HF power components of FHR) significantly increase after propranolol infusion.

### Maternal and fetal heartbeats coupling ratio

As depicted in Table 1, none of the fetal and maternal heartbeats coupling ratios were significantly affected by saline infusion. Atropine, on the other hand, has led to a decrease in CP_heartbeats_ for ratios [1:2] and [1:3], but an increase in [1:4]. In contrast, propranolol has led to a decrease in CP_heartbeats_ for [1:2], and an increase in [1:4] and [1:5]. It is interesting to note that neither saline nor atropine had an effect on CP_heartbeats_ for [1:5].

**Fig. 1.**
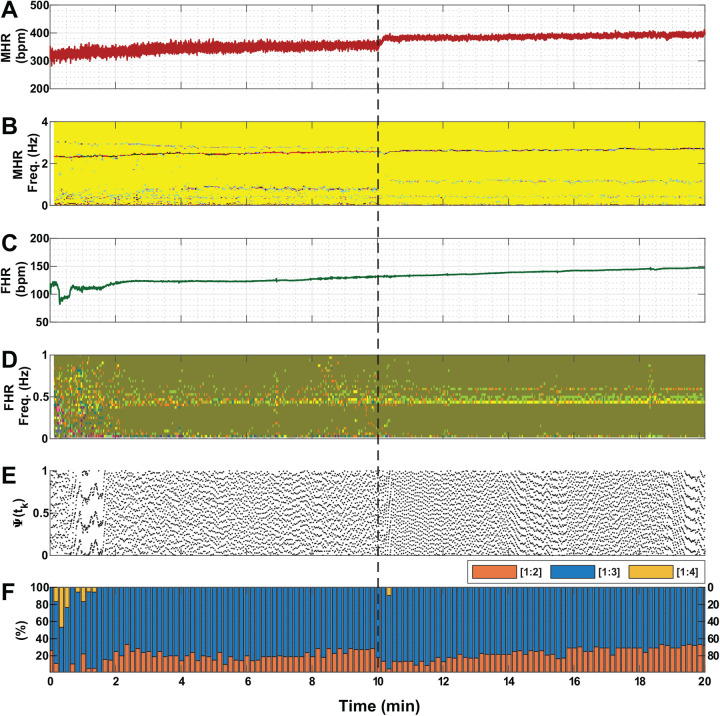
**An example of vital measures of a pregnant mouse and its fetus under saline administration(N_Fetus(Saline)_=14).** The dashed black line indicates the timing of injecting the solution. (A) MHR. (B) Spectrogram of MHR. (C) FHR. (D) Spectrogram of FHR. (E) The instantaneous relative phase (Ψ(*t*_*k*_)). (F) Phase coupling prevalence of different ratios between fetal and maternal heartbeats [*n*:*m*]. In this study, *n* was set to 1 and *m* was varied from 2 to 5. For better clarity, values are displayed for three ratios only (i.e. [1:2], [1:3] and [1:4]) because values for the other two ratios (i.e. [1:5] and [1:6]) are mostly zero. The example shows that saline infusion did not produce a significant change in MHR and FHR. In addition, coupling prevalence of different ratios between fetal and maternal heartbeats were not changed, and the spectrogram did not reveal any major changes in power in the time-frequency map. To elaborate, most powerful frequencies of MHR were found to be clustered within the range of 2–4 Hz, which were not visibly affected by saline infusion. bpm, beats per minute; min, minute.

**Fig. 2.**
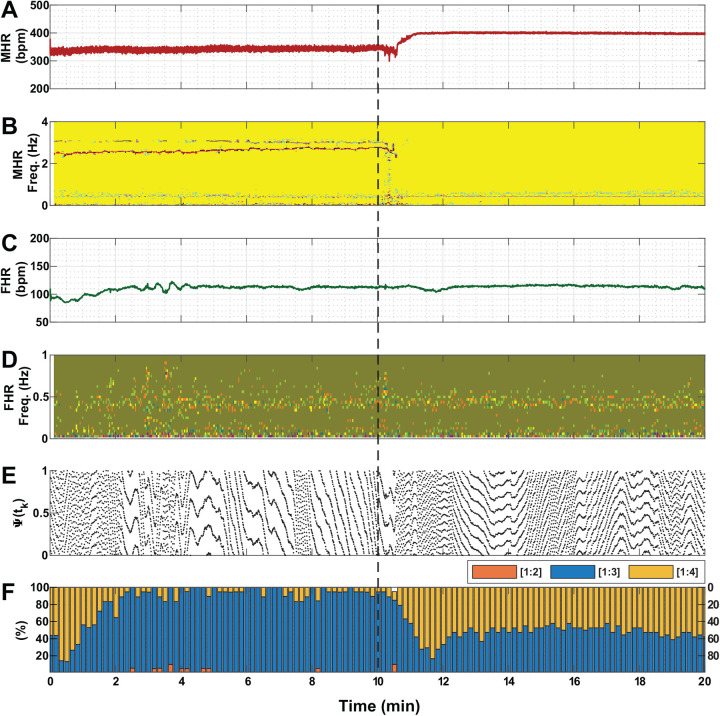
**An example of vital measures of a pregnant mouse and its fetus under atropine administration (N_Fetus(Atropine)_=17).** The dashed black line indicates the timing of injecting the solution. (A) MHR. (B) Spectrogram of MHR. (C) FHR. (D) Spectrogram of FHR. (E) The instantaneous relative phase (Ψ(*t*_*k*_)). (F) Phase coupling prevalence of different ratios between fetal and maternal heartbeats [*n*:*m*]. In this study, *n* was set to 1 and *m* was varied from 2 to 5. For better clarity, values are displayed for three ratios only (i.e. [1:2], [1:3] and [1:4]) because values for the other two ratios (i.e. [1:5] and [1:6]) are mostly zero. The example shows that atropine injection increased the baseline of MHR without any considerable effect on FHR. Moreover, coupling prevalence for heartbeat ratios of [1:2] and [1:3] were decreased, while the prevalence for ratio [1:4] was increased. Furthermore, the spectrogram showed a loss of pre-dominant frequency powers between 2–4 Hz. bpm, beats per minute; min, minute.

**Fig. 3.**
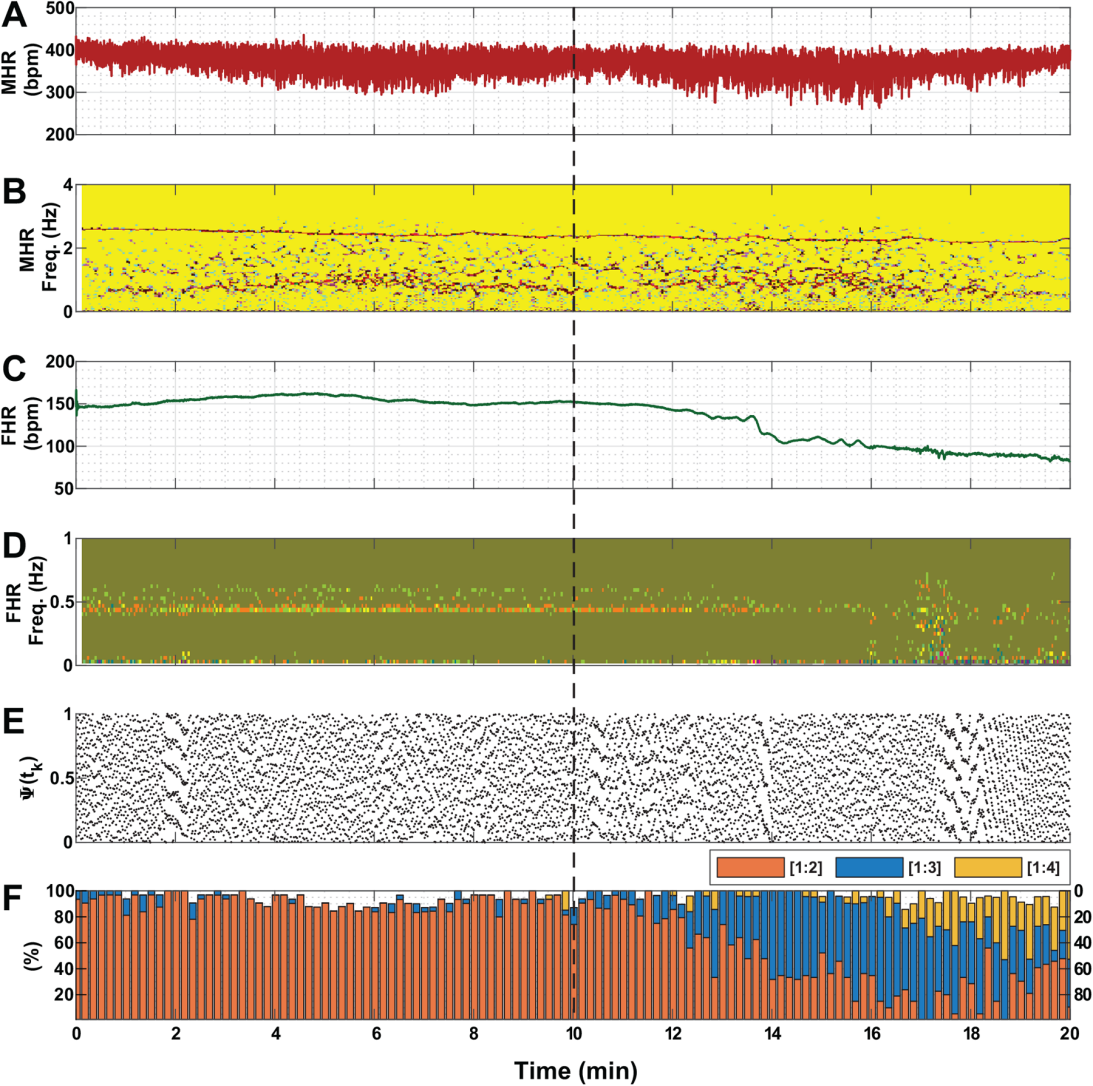
**An example of vital measures of a pregnant mouse and its fetus under propranolol administration (N_Fetus(Propranolol)_=17).** The dashed black line indicates the timing of injecting the solution. (A) MHR. (B) Spectrogram of MHR. (C) FHR. (D) Spectrogram of FHR. (E) The instantaneous relative phase (Ψ(*t*_*k*_)). (F) Phase coupling prevalence of different ratios between fetal and maternal heartbeats [*n*:*m*]. In this study, *n* was set to 1 and *m* was varied from 2 to 5. For better clarity, values are displayed for three ratios only (i.e. [1:2], [1:3] and [1:4]) because values for the other two ratios (i.e. [1:5] and [1:6]) are mostly zero. The example shows that MHR was not affected by propranolol administration. In contrast, FHR slowly decreased, and its spectrogram displayed a wider spread of power between 0.001–0.5 Hz. Moreover, propranolol had led to a decrease in coupling prevalence for ratio [1:2], but an increase for ratios [1:4] and [1:5]. bpm, beats per minute; min, minute.

Each of Figs 1 to 3 shows a representative example associated with the administration of one of the three drug solutions (saline, atropine and propranolol, respectively) in the conducted experiments. For better clarity, percentage values in Panel F of the three figures are displayed for three ratios only (i.e. [1:2], [1:3] and [1:4]) because values for the other two ratios (i.e. [1:5] and [1:6]) are mostly zero. Fig. 1 shows that saline infusion did not produce a significant change in MHR and FHR. Phase coupling prevalence of different ratios between fetal and maternal heartbeats were not changed much under saline administration as compared to atropine and propranolol infusion (Figs 2F and 3F, respectively). Short-term occasional strong coupling prevalence are visible in all cases in this study (Figs 1–3, panel F).

The spectrogram plots shown in Fig. 1 did not reveal any major changes in power in the time-frequency map. To elaborate, most powerful frequencies of MHR were found to be clustered within the range of 2–4 Hz, which were not visibly affected by saline infusion. However, statistically significant (*P*<0.05) observations were found for MHR under atropine administration (Fig. 2); firstly, the loss of pre-dominant frequency powers between 2–4 Hz; secondly, changes in coupling prevalence for most ratios; and lastly, an increase in the baseline. When propranolol was injected (Fig. 3), FHR slowly decreased, and its spectrogram displayed a wider spread of power between 0.001–0.5 Hz. Besides, MHR was not shown to be affected by propranolol administration.

**Fig. 4.**
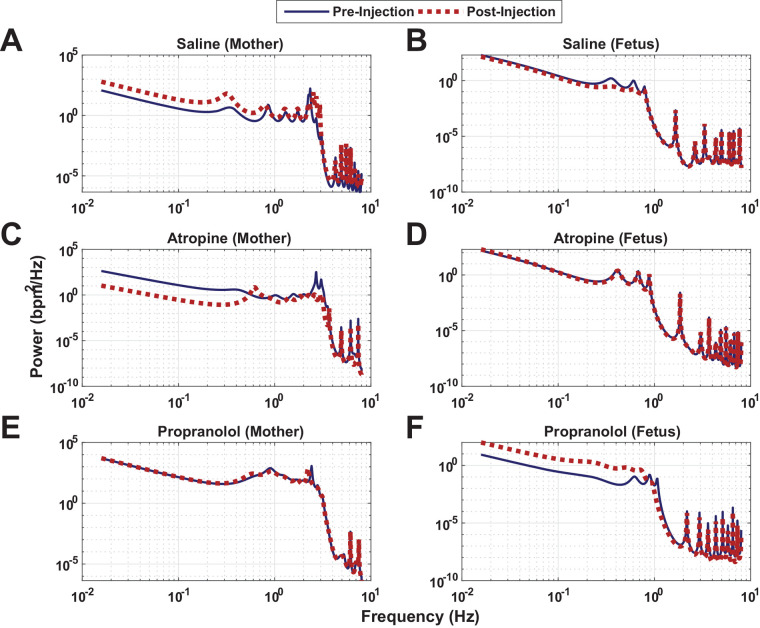
**Examples of PSD of MHR and FHR and the resulting changes induced by saline, atropine and propranolol administration.** The PSD functions of the concerned signals over the pre-administration period of 5 min are shown as solid blue lines. The results for the 5 min duration of post-administration are represented as dotted red lines. (A,B) Saline administration was not shown to affect the PSDs of both of MHR and FHR, respectively. (C,D) The injection of atropine resulted in visible decreases in powers at LF and HF zones of MHR. However, no changes were observed in the PSD of FHR. (E,F) Propranolol infusion was shown to have no changes in PSDs of MHR, but resulted in a visible increase in the power present in FHR for the LF range, respectively. bpm, beats per minute; Hz, hertz.

Fig. 4 shows the PSD of MHR and FHR signals and the resulting changes induced by saline, atropine and propranolol administration. The PSD functions of the concerned signals over the pre-administration period of 5 min are shown as solid blue lines. The results for the 5 min duration of post-administration are represented as dotted red lines. It can be depicted from Fig. 4A,B that saline administration was not shown to affect the PSDs of both of MHR and FHR. However, atropine administration resulted in visible decreases in powers at LF and HF zones of MHR (Fig. 4C). Moreover, no changes were observed in the PSD of FHR after the injection of atropine (Fig. 4D). In contrast, propranolol administration was shown to have no changes in PSDs of MHR (Fig. 4E) but resulted in a visible increase in the power present in FHR for the LF range (Fig. 4F).

## DISCUSSION

This study explored the development of a technique for analyzing mice FHRV and MHRV by using fetal and maternal ECG measurements and their response to pharmacological autonomic blockades. The normative results listed in Table 1 can serve as an imperative benchmark for future studies in this area of research. Fetal and maternal HRV features in time and frequency domains were analyzed under two types of solutions: non-pharmacological (saline), and pharmacological. The later considers parasympathetic (cholinergic) blockades such as atropine (Morris et al., 2007), and sympathetic (adrenergic) blockades such as propranolol (Chien et al., 2013).

The development of cardiac autonomic nervous system in mice commonly starts at E8.5, when the neural crest cells (NCCs) start to migrate from the neural tube, and ultimately reach the dorsal aorta and the outflow tract at E9.5 and E10–E10.5, respectively (Végh et al., 2016). It has been shown that parasympathetic vagal innervation precedes sympathetic innervation (Shoba and Tay, 2000). NCC-derived vagal nerve fibers are observed at the venous pole of the heart at E12.5 and are classified as cholinergic neurons. At E15.5, the sympathetic nerves were reported to reach the apex at the dorsal side of the heart. It can thus be speculated that fetal mice of E17.5 would have an autonomic system that responds to the effects of atropine and propranolol administration. In this study, therefore, fetuses of this particular gestational age (i.e. E17.5) were used based on the discussion above.

### HRV is a measure of autonomic modulation in mice and its fetuses

This study demonstrates that quantitative characterization of autonomic nervous system modulation of beat-to-beat HR regulation is technically feasible in anesthetized pregnant mother and fetal mice. Furthermore, two major PSD components [LF (0.001–1 Hz) and HF (2–4 Hz) ranges] were observed based on the conducted experiments. Alterations in the LF component in fetal mice were regulated by the sympathetic nervous system because propranolol administration was shown to change the LF band of FHR. However, HF components of MHR (power in the frequency range of 2–4 Hz), which are predominantly vagally mediated as revealed by atropine administration, were reduced significantly. Mice HR regulation for the frequency power in the range of 2–4 Hz could possibly be related to parasympathetic nervous system activity as analogous to that of human HR regulation, apart from being ten times higher (note that the HF range of humans is 0.15–0.4 Hz; Task Force of the European Society Organization Electrophysiology, 1996). As MRMSSDHR was decreased by the blockade of parasympathetic nerve activity, the corresponding MMHR was increased. This phenomenon might indicate that fetal mice show antagonistic response to the vagal inhibition. The application of standard HRV and the technique of phase coupling analyses, coupled with study protocols for investigating the effects of maternal conditions on fetal mice will help identify the role of the autonomic nervous system in HR regulation in fetal mice models.

### Role of placental transmission

We showed in this study that propranolol treatment has affected FHRV features, whereas the injection of atropine had little effect on FHR. A previous study on unanesthetized pregnant ewes reported a similar finding: the administration of atropine in the placenta were reported to increase MHR by 25% without changing maternal or fetal arterial pressure and FHRV (Murad et al., 1981). Furthermore, it is reported that atropine treatment increased FMHR in pregnant human (Onnen et al., 1979). It is thus necessary to consider differences in the structure of the placenta for each animal species. Some articles showed that due to the high lipid solubility of propranolol, it passed the placental barrier rapidly in rat (Watanabe et al., 1990), sheep (Marshall et al., 1981) and human (Schneider and Proegler, 1988). In our study, the results demonstrated the decrease in FHR by the administration of propranolol on pregnant mice. It can thus be speculated that the transmission of propranolol through the placental barrier has caused this change. On the other hand, FSDNNHR along with LF and HF power components of FHR were significantly increased by injecting mother mice with propranolol. Since the acute propranolol injection reduced both myometrial and placental blood flow by 45% and 50%, respectively (Karlsson et al., 1984), FHR and fetal autonomic parameters could possibly be affected by this reduction of blood flow.

### Fetal-maternal heartbeats coupling ratio

The administration of autonomic blockades had more effect on fetal-maternal heartbeats coupling within one fetal cardiac cycle (i.e. *n*=1) compared to saline infusion. The decrease in CP_heartbeats_ for ratios [1:2] and [1:3], in addition to the increase in [1:4] and MHR by atropine (Table 1) strongly confirms that maternal vagal nervous system works in the range of two and three beats within one fetal heartbeat. On the other hand, the increase in CP_heartbeats_ for [1:4] and [1:5] by propranolol, along with the decrease in FHR could indicate that the fetal sympathetic nervous system acts on a higher scale to match four and five maternal heartbeats. There are no previous works on fetal-maternal heartbeats coupling in mice. However, evidence of coupling events between the heart rates on beat-by-beat was previously reported for human fetuses (Ivanov et al., 2009; Riedl et al., 2009; Van Leeuwen et al., 2003). The short-term occasional coupling was found in Van Leeuwen et al. (2013) by using phase synchronization analysis as the phase locking of the rhythmic maternal and fetal heartbeats. The short-time fetal-maternal heartbeats coupling might be occurring via mechanical or auditory stimuli associated with the maternal rhythms, perceived by the fetus as previous studies suggested (Riedl et al., 2009; Van Leeuwen et al., 2009). However, the actual determination of the underlying physiological mechanisms needs further investigation.

### Effect of anesthesia

In this study, pregnant mice were anesthetized with a mixture of ketamine and xylazine, which is one of the most commonly used anesthetics in animal studies. Isoflurane was used in addition to ketamine/xylazine to maintain the anesthesia. Propranolol administration was not found to have decreased MHR in this study. This could partially be due to the effect of ketamine/xylazine anesthesia, which was previously reported to reduce the HR in isolated rat heart preparations (Aronson and Hanno, 1978), and also *in vivo* conditions (Brown et al., 1994; Maignan et al., 2000; Sapru and Krieger, 1979). In addition to that, isoflurane was reported to reduce the HR in mice (Tan et al., 2003). Ketamine/xylazine induces elongation of R-R and QT intervals (Aronson and Hanno, 1978), a decrease of blood pressure (Sapru and Krieger, 1979), as well as a reduction of renal sympathetic activity and attenuation of baroreflex sensitivity *in vivo* (Akine et al., 2001). The echocardiography study also revealed that both β-agonist isoproterenol and atropine had led to an increase in HR and fractional shortening in ketamine/xylazine anesthetized mice. In contrast, the cardiac function was unchanged by the β_1_-antagonist atenolol in anesthetized mice (Tan et al., 2003). On the other hand, the increase in MHR followed by atropine administration did not seem to be affected by the influence of ketamine/xylazine anesthesia, which was reported to increase parasympathetic activity and suppress sympathetic and baroreceptor activity, resulting in marked bradycardia (Švorc et al., 2013).

The proposed approach could be useful for physiological assessment of the impact of maternal autonomic modulation activity on fetal distress or compromises, such as cardiomyopathies, long QT syndromes, pre-eclampsia, and other forms of complications prevalent in pregnant mothers. The HR of large animals, including humans and rabbits, decreases through development (Adachi et al., 2013); whereas the HR of mice increases during prenatal development until E15.5, and then decreases till birth (Khandoker et al., 2016). This could imply that HR regulation in fetal mice is due to the sympathetic nervous system until E15.5, after which cardiac parasympathetic activity primarily regulates the HR as the vagal tone develops before birth. These differences in maturation of cardiac innervation and pathways should be considered when comparing fetal mice to humans. Further research is warranted to elucidate the process of developing autonomic nervous system from early to late fetuses. Further studies should explore the effects of different doses of the autonomic antagonists.

## Conclusions

This article presented an experimental investigation to examine the role of autonomic control of maternal and fetal HRV in time and frequency domains, along with their heartbeats phase coupling prevalence in pregnant mice models (E17.5) by blocking cholinergic and β-adrenergic nerve activities with atropine and propranolol, respectively. Various HRV features and coupling prevalence for different ratios were calculated from the recorded maternal and fetal ECG signals preceding and following the injection of each solution into an individual mother.

Results demonstrated that saline infusion did not produce any significant changes in maternal and fetal HRV as well as coupling prevalence. However, the administration of atropine increased MHR and decreased its variability significantly without any considerable effect on FHR. In addition, it significantly reduced the HF power component of MHR. Moreover, coupling prevalence for fetal:maternal ([*n*:*m*]) heartbeat ratios of [1:2] and [1:3] were decreased due to atropine, while the prevalence for ratio [1:4] was increased. Propranolol infusion did not produce any significant changes in MHR and its variability; however, it significantly decreased FHR and increased FHRV. In addition, the sympathetic nervous system regulated the alterations in the LF component of FHR. Moreover, propranolol had led to a decrease in coupling prevalence for ratio [1:2], but an increase for ratios [1:4] and [1:5].

The purpose of this research has been clarified and validated by qualifying the application of standard HRV and the technique of phase coupling analyses, coupled with study protocols for investigating the effects of maternal conditions on fetal mice to identify the role of the autonomic nervous system in HR regulation in fetal mice models. This is vital for understanding the nervous system in general and assessing the underlying impact of maternal autonomic modulation activity on fetal health and pregnancy complications in particular.

## MATERIALS AND METHODS

### Mice subjects and experiment protocol

A total of 48 mice fetuses from 27 female mice of type *C57BL/6J* were used in this study. Mice were housed in cages under control lighting of 12:12 h light-dark cycle and temperature of (22±2°C). Table 2 summarizes the sample size and weight mean±standard deviation (SD) of pregnant mice and fetuses used in the experiments at E0 and E17.5 (i.e. prior and during gestation, respectively). It is noteworthy that aseptic conditions were maintained throughout the experiments. Pregnant mice were anesthetized with subcutaneous ketamine (Ketalar 500 mg Daiichi-Sankyo: 100 mg kg^−1^) and xylazine (Rompun injection solution 2% Bayer; 10 mg kg^−1^), and maintained with inhalational isoflurane (Forane AbbVie Inc. 0.5%, 260 ml min^−1^). The experimental protocol for this study was approved by the Tohoku University Committee for Safety Management of Animals. Additionally, all experiments were performed in accordance with relevant guidelines and regulations approved by the Tohoku University School of Medicine.

**Table 2.**
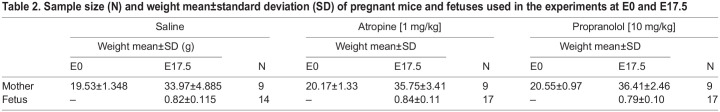
Sample size (N) and weight mean±standard deviation (SD) of pregnant mice and fetuses used in the experiments at E0 and E17.5

**Fig. 5.**
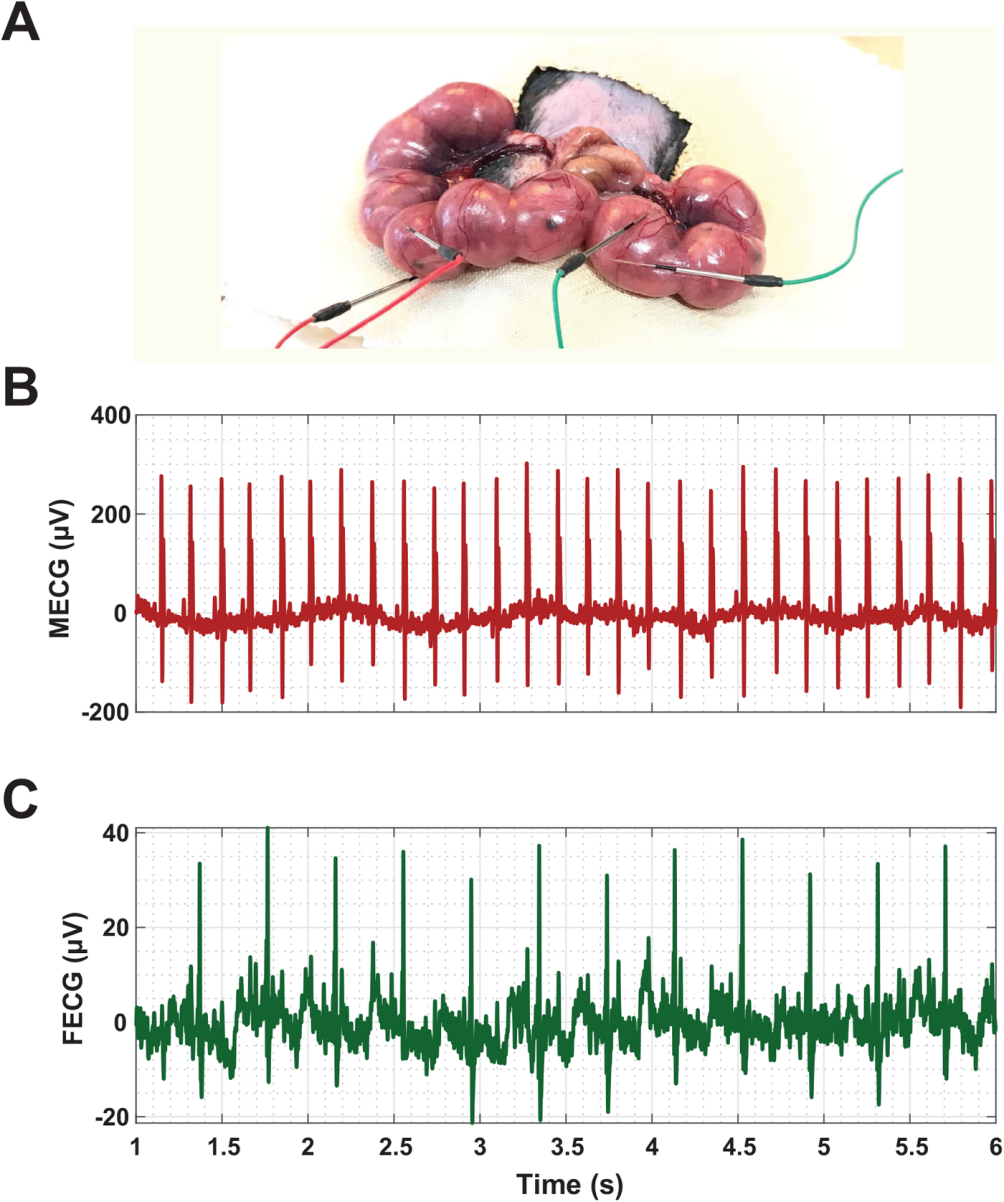
**Experimental setup of the fetal electrocardiography system for embryonic mouse, and examples of maternal and fetal electrocardiogram (ECG) signals from anesthetized pregnant mouse.** (A) The experimental setup of the fetal ECG recording system in which pregnant mice underwent surgery at embryonic day (E)17.5 of gestation. (B) Maternal ECG (MECG). (C) Fetal ECG (FECG). µV, micro-Volt; s, seconds.

The fetal electrocardiography system for embryonic mouse was explained in our previous studies (Khandoker et al., 2016, 2017). Fig. 5 shows the experimental setup and examples of fetal and maternal ECG signals recorded during the experiment. At E17.5, ECG signals of pregnant mice and their fetuses were simultaneously recorded by using a biomedical amplifier and recording system (Polymate AP1532; TEAC Co., Tokyo, Japan) at a sampling frequency of 1 kHz. After 10 min of ECG signals recoding, separate solutions of saline [100 μl (10 g)^−1^ body weight (BW)], atropine [1 mg kg^−1^ in 100 μl (10 g^−1^) BW], and propranolol [10 mg kg^−1^ in 100 μl (10 g^−1^) BW] were injected into individual mothers' interscapular subcutis at 37°C. Following the injections, the recording was continued for another 10 min.

### Time domain MHRV and FHRV

HRV features (Task Force of the European Society Organization Electrophysiology, 1996) including the mean value of maternal or fetal HR (MMHR, FMHR), the standard deviation of NN intervals in maternal or fetal HR (MSDNNHR, FSDNNHR), and the square root of the mean squared differences of the successive normal maternal or fetal heartbeats (MRMSSDHR, FRMSSDHR) were calculated from RR intervals during the last 5 min recording of the ECG signals prior to drug administration (i.e. pre-injection). Likewise, HRV features were computed for the last 5 min duration prior to the end of the recordings after the solutions have been injected (i.e. post-injection).

### Frequency domain MHRV and FHRV

In order to analyze and examine whether the frequency content of local sections of the ECG signals were affected by the infusion of saline, atropine, or propranolol, Short-time Fourier transform was applied on MHR and FHR time series (recorded for 20 min). A kaiser window of 256 samples was used to segment the data with 50% overlap in order to calculate the spectra. Power spectral analysis was then performed on resampled (16 Hz) time series of 5 min clips of MHR and FHR at pre- and post-injections using Burg method with an order of 30 (Kazlauskas, 2011). The 1012 point Fast Fourier transform (FFT) was repeatedly computed with 50% overlap between adjacent segments. The power spectral of each segment was then computed and averaged. Subsequently, the power spectral density (PSD) analysis was estimated by numerical integration over the frequency ranges of 0.001–1 Hz and 2–4 Hz. These spectra were denoted as the low and high frequency (LF and HF, respectively) bands, respectively.

### Phase coupling

Phase coherence method was used to investigate the heartbeats phase coupling prevalence (CP_heartbeats_) between R-peaks of fetal and maternal ECG signals as described in a previous article (Niizeki and Saitoh, 2014). The instantaneous phase time series was obtained by:
(1)

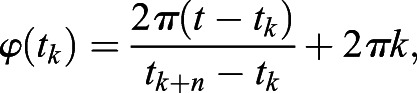
where *t* and *t*_*k*_ are the timings of R-peaks of maternal and fetal ECG signals, respectively, and *n* is the number of fetal heartbeats. The instantaneous relative phase (Ψ(*t*_*k*_)) over the time window of *t*_*w*_ with respect to fetal ECG signal was calculated using the following equation:
(2)

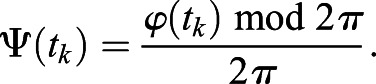
The fetal:maternal phase coupling ratio ([*n*:*m*]) with *n*=1 was defined by:
(3)

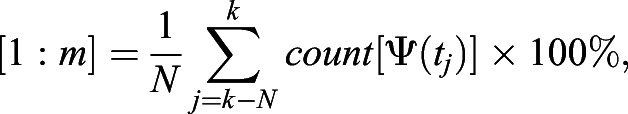
where *N* is the number of heartbeats in time window of *t*_*k*_−*t*_*w*_/2≤*t*_*j*_<*t*_*k*_+*t*_*w*_/2. In this study, *m* ranges from 2 to 5 for maternal heartbeat synchronization within each fetal cardiac cycle. Additionally, *t*_*w*_ was chosen to be 10 s.

**Fig. 6.**
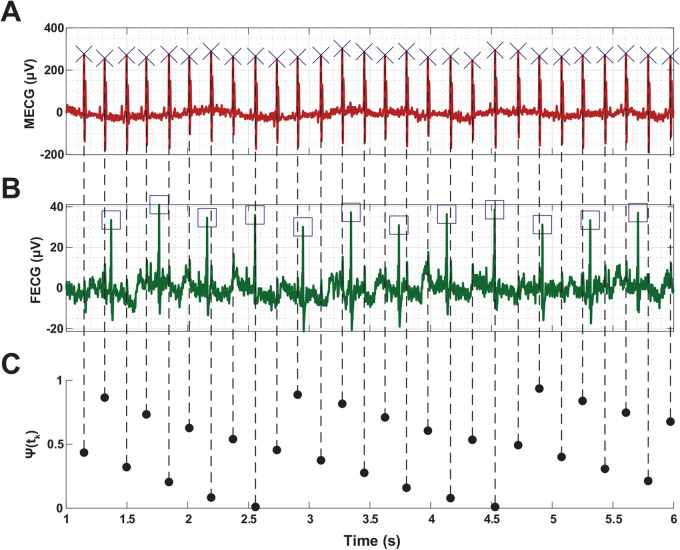
**An example on maternal-fetal phase coupling pattern derivation from the timings of the R–peaks of the MECG signal with respect to the same of the FECG signal.** (A) MECG signal. (B) FECG signal. (C) The example demonstrates a representation for estimating the instantaneous relative phase (Ψ(*t*_*k*_)) – defined as the normalized angle of MECG relative to the start of FECG – and the corresponding phase coupling when considering a fetal:maternal ([*n*:*m*]) ratio of [1:2]. Within one fetal cardiac cycle, Ψ(*t*_*k*_) shows two different values that correspond to ratios [1:2] and [1:3]. To elaborate, two parallel lines are shown in the synchrogram for the total duration, except for the time periods 2.5–3 s and 4.5–5 s in which three lines appear corresponding to a synchronization ratio of [1:3]. µV, micro-Volt; s, seconds.

Fig. 6 shows an example on how to estimate Ψ(*t*_*k*_) and the corresponding phase coupling when considering an [*n*:*m*] ratio of [1:2]. Within one fetal cardiac cycle, Ψ(*t*_*k*_) shows two different values that correspond to ratios [1:2] and [1:3]. To elaborate, two parallel lines are shown in the synchrogram for the total duration, except for the time periods 2.5–3 s and 4.5–5 s in which three lines appear corresponding to a synchronization ratio of [1:3].

### Statistics

Data are presented as mean±s.d. in Tables 1 and 2. Comparisons between pre- and post-injection values were done by a Wilcoxon's signed rank test (Gibbons and Chakraborti, 2011). Differences were considered significant at *P*<0.05.

Prior to drug administration (saline, cholinergic and β-adrenergic blocking agents), the mean values of HRV and CP_heartbeats_ for four different ratios ([1:2], [1:3], [1:4] and [1:5]) were calculated over a 5 min sample period. Likewise, the same statistics were calculated right after drugs injection over a 5 min duration. In order to determine the magnitude of the effect of saline and autonomic blocking agents, Cohen's *d* effect statistic along with its 95% confidence interval were also calculated (Nakagawa and Cuthill, 2007; Végh et al., 2016) to provide the difference in mean values between two different treatments in units of pooled s.d. The magnitude of effect sizes to saline and the other considered drugs in this study was categorized to be either small (0.2≤|*d*|<0.5), moderate (0.5≤|*d*|<0.8), large (0.8≤|*d*|<1.2), or very large (|*d*|≥1.2) (Nakagawa and Cuthill, 2007).
